# Computer-Assisted Analysis of Microplastics in Environmental Samples Based on μFTIR Imaging in Combination with Machine Learning

**DOI:** 10.1021/acs.estlett.1c00851

**Published:** 2021-12-09

**Authors:** Benedikt Hufnagl, Michael Stibi, Heghnar Martirosyan, Ursula Wilczek, Julia N. Möller, Martin G. J. Löder, Christian Laforsch, Hans Lohninger

**Affiliations:** † Institute of Chemical Technologies and Analytics, Vienna University of Technology, A 1060 Vienna, Austria; ‡ Purency GmbH, Walfischgasse 8/34, A 1010 Vienna, Austria; § Department of Animal Ecology I and BayCEER, University of Bayreuth, D 95 440 Bayreuth, Germany

## Abstract

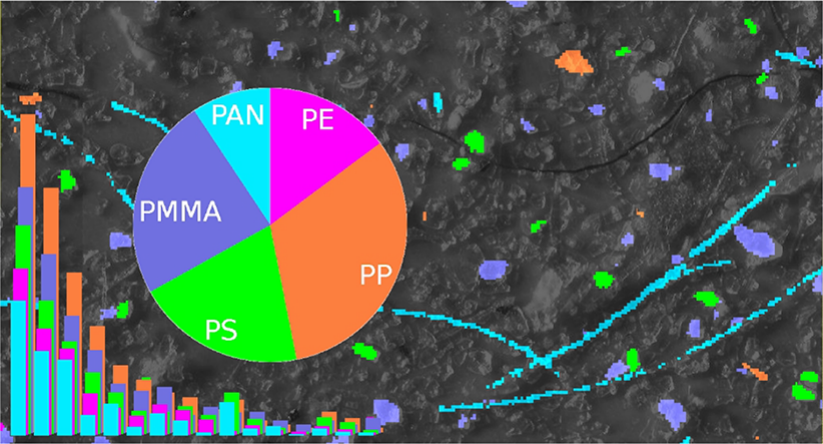

The problem of automating the data analysis of microplastics following a spectroscopic measurement such as focal plane array (FPA)-based micro-Fourier transform infrared (FTIR), Raman, or QCL is gaining ever more attention. Ease of use of the analysis software, reduction of expert time, analysis speed, and accuracy of the result are key for making the overall process scalable and thus allowing nonresearch laboratories to offer microplastics analysis as a service. Over the recent years, the prevailing approach has been to use spectral library search to automatically identify spectra of the sample. Recent studies, however, showed that this approach is rather limited in certain contexts, which led to developments for making library searches more robust but on the other hand also paved the way for introducing more advanced machine learning approaches. This study describes a model-based machine learning approach based on random decision forests for the analysis of large FPA-μFTIR data sets of environmental samples. The model can distinguish between more than 20 different polymer types and is applicable to complex matrices. The performance of the model under these demanding circumstances is shown based on eight different data sets. Further, a Monte Carlo cross validation has been performed to compute error rates such as sensitivity, specificity, and precision.

## Introduction

Although microplastics (MPs) are omnipresent in nature, their impact on environmental and human health remains widely unclear. Since the impact of microplastics on ecosystem functions, as well as on organisms, depends on the exposure level and the material properties of the particles, it is indispensable to accurately evaluate the microplastic contamination with regard to polymer type, shape, and size. Therefore, appropriate analytical tools and methods need to be found since visual identification is extremely prone to bias with error rates up to 70%.^1^

The analysis of microplastics by means of spectroscopy^2,3^ is one of the most widely used technologies, since it allows the identification of particles based on characteristic vibrational bands. The investigation of microplastics in the micrometer range requires the processing of samples to isolate and concentrate the miroplastic particles on filters. Since nonplastic particles still remain on the filter as well, sequential single point measurements of the spectra of each particle is extremely time consuming when targeting the whole surface of a filter.

A solution to this problem is focal plane array (FPA)-based micro-Fourier transform infrared (FTIR) imaging which facilitates the generation of chemical images by simultaneously recording several thousand spectra within one single time-saving measurement.^4^ However, manual comparison of the received spectra with the reference spectra is extremely time consuming and not suitable for monitoring studies where many samples need to be analyzed. Hence, regardless of whether the measurement of microplastics is based on Fourier-transform infrared (FTIR), quantum cascade laser (QCL), or Raman spectroscopy, usually the necessesity arises to process the spectral information automatically.

Thus, a broad range of algorithms already exists that have been applied to the task of computer-assisted analysis of spectroscopic MP data. These can be divided into *model-based*(5−11) and *instance-based*(12−20) machine learning approaches. Model-based approaches first infer a statistical model from spectroscopic reference data. The model is then applied to unknown spectra to assign them to predefined classes, which can be anything from a polymer type or a matrix component. On the other hand, instance-based approaches directly apply the spectroscopic reference data, in this case the “instances”, for identifying unknown spectra by means of similarity measures. From the viewpoint of analytical chemistry, the latter approaches represent the well-known spectral library search engines where *hit quality indices* (HQIs) are computed by means of different measures such as the Pearson correlation coefficient.

Both types of machine learning have their strengths and weaknesses. Instance-based learning comes with the advantage that the spectroscopic reference data can be enhanced or adapted with relative ease just by changing the reference spectra in the library. Model-based learning usually requires a high degree of chemometric expert knowledge which makes application specific changes more difficult. However, regarding analysis speed, model-based machine learning clearly outperforms the latter. Primpke et al.^21^ benchmarked two instance-based algorithms on a data set consisting of 1.8 million spectra. The analysis time ranged from 4 to 48 h. On the other hand Hufnagl et al.,^9^ who applied a model-based learning approach, reported an analysis time of about 5 min based on 1 million spectra for detecting five different polymers types. As typical data set can be as large as 25 GB, which corresponds to 5 million spectra, these differences may have strong implications regarding the applicability of such algorithms in the context of high-throughput monitoring analysis.

This letter describes a model-based machine learning approach based on random decision forest (RDF) classifiers^22^ for analyzing FPA-based μFTIR hyperspectral images. Hufnagl et al.^9^ described the preliminaries to derive such models for five different polymers. In this study, we focus on an extended version of the model which can already detect more than 20 different polymer types (Table 1) including the 10 most important polymers with respect to the production volume.^23^ Compared to other model-based learning approaches^10,11^ for FPA-based μFTIR imaging, the herein described model has the broadest applicability in terms of number of polymers. Further, it has been trained to be applicable to different matrices such as air, water, soil, and sewage sludge. In this study, we also show analysis results for different environmental samples and validate the RDF model by means of Monte Carlo cross-validation.^24,25^ Complete views of the experimental data and additional tables summarizing performance measures can be found in the Supporting Information (SI).

**Table 1:** Supported Polymer Types and Performance Measures^26^ for Respective Classes

Systematic name	Abbreviation/Class ID	Sensitivity	Specificity	Precision
polypropylene	PP	0.957 1	0.998 4	0.971 0
polyethylene	PE	0.978 5	0.998 5	0.974 0
polyvinyl chloride	PVC	1.000 0	0.999 6	0.979 6
polyurethane	PU	0.967 2	0.999 2	0.970 2
polyethylene terephthalate	PET	0.982 4	0.998 9	0.975 7
polystyrene	PS	0.981 9	0.999 4	0.979 2
acryl butadiene styrene	ABS	0.986 1	0.999 9	0.994 4
polyamide	PA	0.957 5	0.999 1	0.979 7
polycarbonate	PC	0.970 6	0.999 6	0.970 6
poly(methyl methacrylate)	PMMA	0.982 7	0.999 3	0.982 7
cellulose acetate	CA	1.000 0	0.999 9	0.993 4
ethylene vinyl acetate	EVAc	0.973 7	0.999 8	0.989 3
ethylene vinyl alcohol	EVOH	0.977 9	0.999 1	0.970 8
polyacrylonitrile	PAN	0.946 7	1.000 0	0.996 5
polybutylene terephthalate	PBT	0.982 5	0.999 5	0.970 4
polyether ether ketone	PEEK	0.936 1	0.999 5	0.965 6
polyoxymethylene	POM	0.953 3	1.000 0	0.996 5
polyphenylsulfone	PPSU	0.964 7	0.999 4	0.956 3
polysulfone	PSU	0.970 0	0.998 8	0.912 2
silicone	silicone	0.925 0	0.999 9	0.988 5
polylactic acid	PLA	0.986 5	0.999 4	0.981 2
	Other	0.981 4	0.979 2	0.977 4

## Materials and Methods

### Sample Purification and Preparation

The main difficulty in analyzing MPs in environmental water samples is that their abundances are usually very low with respect to the sample volume. Due to this fact, a sample concentration is necessary which also leads to a concentration of other seston particles in excess. Thus, purifications of the concentrated MP samples are mandatory before hyperspectral imaging can be applied.

Renner et al.^27^ and Möller et al.^28^ provide an overview of different sample preparation schemes which have been reported in the literature. It is important to ensure that MPs do not degrade, or worse, be lost entirely during that process. Depending on the polymer type, the reagents used, and the temperature, as well as the exposure time, particle surface properties and chemistry as well as sizes may change, which thus biases the analysis result. Hurley et al.^29^ highlighted this issue by comparing three different protocols either using oxidative digestion, Fenton’s reagent, or alkaline digestion for preparing sewage sludge and soil samples.

In this study, the samples have been prepared following the methodology described by Löder et al.^30^ This protocol applies multiple enzymatic digestion steps in order to remove most of the biological matrix. The sample is then filtered through an aluminum oxide filter (Anodisc 0.2 μm pore size, 10 mm diameter) which is the sample carrier for the spectroscopic analysis. As this procedure avoids the use of strong acidic or alkaline solutions, the MPs are preserved in their original states. A short summary of the procedure is given in the SI.

### FTIR Imaging

The herein presented FPA-based μFTIR images have been measured using a Bruker Hyperion 3000 FTIR imaging microscope and a Bruker Lumos II FTIR imaging microscope (www.bruker.com). The Hyperion 3000 is equipped with a 64 × 64 pixel FPA detector coupled to a Tensor 27 spectrometer. Each pixel has a size of approximately 11 μm × 11 μm. In the spectral domain, the images cover a range between 1250 and 3595 cm^–1^ at a resolution of 4 cm^–1^ More details and a discussion regarding the measurement setup can be found in Löder et al.^4^ as well as Hufnagl et al.^9^ The Lumos II uses a 32 × 32 FPA detector and has a built-in FTIR spectrometer. For the subsequent chemometric analysis, the FTIR images are exported from the instrument software Bruker Opus using the widely used ENVI format.

### Multiclass Modeling and Training Data Design

The computer-assisted identification of MPs by means of classification using the above equipment and measuring conditions comes with many different challenges. Even though sample purification procedures will remove most of the biological matrix, usually some residual bio-organic compounds remain that exhibit characteristic vibrational bands very similar to polymers. The particle size further induces two additional problems. Small particles will diffract the IR radiation if their size draws near to the electromagnetic wavelengths of the illumination source causing (resonant) Mie scattering. This effect distorts baselines and in the more severe case a shifting of peak positions as well as peak deformations.^31^ On the other side, if particle thickness reaches a point where certain wavelength ranges are fully absorbed, the total absorption (TA) effect shifts relative peak ratios and in the extreme case destroys all information required for the identification. Weathering of polymers, as well as the presence of additives and pigments, may also change peak patterns, which is another issue that may cause a classification error.

In order to create a basic set of spectral references for training the RDF, spiked samples containing mixtures of the 21 different polymers were created. Some polymers where already available as powders while others were obtained through abrasion from a larger polymer material. This initial set of hyperspectral images (HSIs) was then labeled by four independently working experts to establish a ground truth for the 22 classes. Possible label noise^32^ was reduced by creating four independent RDF models based on the respective expert data sets. These were then applied to the training data of the other experts to indicate possible instances where labeling errors have been made. Label noise is also discussed more in-depth in Hufnagl et al.,^9^ where the effect is illustrated by confusion matrices. The audited data sets were then combined into a basic training data set.

It quickly turned out that the initial model inferred from the basic training data set performed poorly with respect to the target group of μFTIR images mainly because of the large matrix diversity, weathered polymers, and particles that exhibited much stronger TA than present in the training data. To improve the performance with respect to matrix and weathering effects, a large collection of spectra from a variety of sampling sites and matrix types (water, sediment, soil, compost, sewage sludge) was added to the training data. Regarding the TA effect, additional HSIs where taken from larger polymer particles to sample spectra across a broader TA range. In total, the final data set consisted of about 12,000 reference spectra, one-half representing MPs while the other half represented matrix spectra.

### Statistical Performance Assessment

The statistical performance of the RDF classifier was assessed by means of a special form of cross validation (CV) known as Monte Carlo CV.^24,25^ CV is a broadly applied approach for optimization and validation of machine learning models^33^ and has already been applied to validate the model by Weisser et al.^11^ In Monte Carlo CV, which is a nonexhaustive form of CV, multiple training and test data set pairs are created by resampling the spectral reference data according to a splitting ratio. Each data set pair is used to infer an RDF model from the training data which is then applied to the corresponding test data set to compute correct and wrong predictions. By repeating this process over multiple training and test data set pairs, it is possible to summarize the results as a confusion matrix, which is illustrated in Figure S1. Table S2 further shows the original confusion matrix as a table without normalization applied. On the basis of the confusion matrix class specific performance, measures such as *sensitivity*, *specificity*, and *precision* or global measures such as *accuracy* and *Cohen’s kappa* can be computed.^26^

In our setting, we produced 20 random splits where 10% of the reference spectra was used as test data. Table 1 lists the class-specific performance measures, where “Other” denotes the classifier which detects the matrix and the filter. Using a selection of global measures, we computed an *accuracy* of 0.976 6 and a *Cohen’s kappa* of 0.969 0. The accuracy is slightly higher than Cohen’s kappa because in cases where classes are unbalanced (the “Other” class makes up about 50% of the data), the value is biased toward the larger classes. Ballabio et al.^26^ provide an in-depth discussion about the behavior of these measures as well as reference code implementations. Additional global measures are given in Table S1.

### Computer-Assisted Data Analysis

The groundwork for the RDF classifier was laid by Breiman^22^ in 2001 and is based on earlier works of the random subspace method^34^ and bootstrap aggregation^35^ (bagging). Since then, the RDF algorithm has been applied to a variety of machine learning problems^33,36^ and is available in software libraries such as *scikit-learn*(37) or *WEKA*.^38^

In this study, we used the imaging software Microplastics Finder (www.purency.ai), which is based on the Epina ImageLab Engine (www.imagelab.at). The software already implements an RDF classifier in combination with various chemometric tools for particle detection and characterization. By using a built-in scripting engine, we customized the software by developing an add-on which streamlines the application toward MP detection. We dubbed this add-on the Bayreuth Microplastics Finder (BMF) and built a workflow which is depicted in Figure S12.

After importing and calibrating the FTIR image by means of the ENVI import function, the data is analyzed in four steps:

(1) **Detection of the filter substrate**. As the pixels covering the filter substrate contain no spectral information due to background correction, they can be detected statistically. Before the machine learning model is applied, these pixels are excluded from further analysis.
(2) **Classification of the remaining pixels**. In this step the RDF uses the spectral information on each pixel of the HSI and assigns it to one of the 22 classes.
(3) **Postprocessing of the classification**. The original model output is postprocessed by means of different lateral operators so that the information gained from neighboring pixels can be used to further improve the result.
(4) **Particle detection and characterization**. In this final step, particles are detected on the basis that neighboring pixels have to be of the same polymer class and have to be connected over by an edge. In this way, all MPs of the image are detected and stored in the form of a list where each particle receives a unique ID. Further, each particle is characterized using different geometric properties such as length, width, aspect ratio, area, and orientation in addition to a value that describes the reliability of the classification.

The final outcome after the particle detection and characterization is shown in Figure S11 which includes the list of individual particles and the list of total particle counts per class. On top of the visual image, MPs of the respective classes are highlighted in different colors.

Once the analysis process is finished, the user may interactively assess and evaluate the list of detected MPs in the particle editor which is also part of the software package. This can be done by comparing the average spectrum of each list entry with a reference spectrum of a database which is selected based on the detected polymer type. Optionally, the user may choose to manually edit particles by adding or removing pixels.

Finally, the MP list can be exported as a CSV file which allows the user to postprocess and visualize the results in a software of their choosing. CSV can be imported in many software packages including MS Excel, Matlab, and SPSS Statistics to name just a few.

### Application Examples

**Figure 1:**
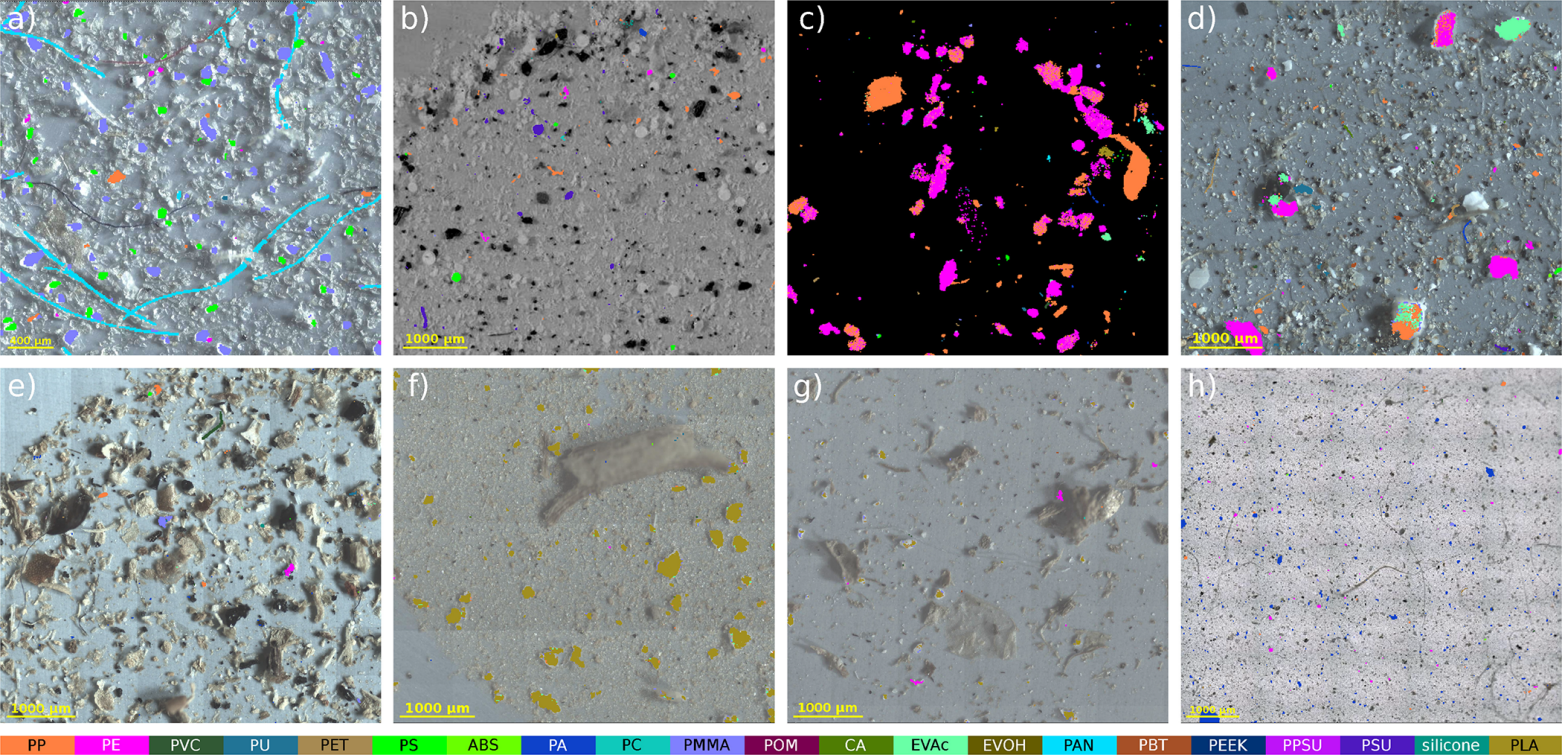
Application examples for different matrices. (a) Plankton sample adapted with permission under a Creative Commons Attribution 3.0 Unported License from Hufnagl et al.^9^ Copyright 2019, The Royal Society of Chemistry, original microscope image superimposed with new classification result. (b, c) Reference samples adapted with permission under a Creative Commons Attribution 4.0 International License from Primpke et al.^15^ Copyright 2018, Springer Nature, original microscope image superimposed with new classification result. Also (d) wastewater treatment plant outlet, (e) deep sediment sample, (f) soil sample, (g) compost sample, and (h) sea salt sample measured with Bruker LUMOS II.

Figure 1 depicts close-up views of a collection of eight samples from different matrices in order to show the broad applicability and robustness of the RDF model for various environmental application scenarios. Figure 1a–c represent well-studied data sets from the literature. See for example Hufnagl and Lohninger^39^ and Wander et al.^40^ for comparison. Figure 1h represents a sea salt sample which was measured using a Bruker Lumos II. All other data sets have been measured using a Bruker Hyperion 3000. Complete views of the filters are available in Figures S3–S10.

Without applying any filter substrate detection, the classification of an image of 1000 × 1000 pixels requires about 20–25 min assuming 20 polymer classes (see Hufnagl et al.^9^ for experimental details and used hardware). This computation time can be reduced to less than 10 min by using the above-mentioned statistical detection technique to exclude pixels from the background for the following reasons. As can be seen in Figures S3– S10, the samples’ particles will cover only a small circular portion of the filter surface. As the measured FTIR image is rectangular, the particles therefore usually cover less than 50% of all the pixels. By excluding the pixels which can be attributed to the background, a significant reduction of computation time can thus be achieved.

## Results and Discussion

### Dual Control

As described in the previous section, the BMF approach employs a dual control or four-eye principle which we recommend due to problems that may arise from sample preparation and data acquisition:

- Even though their concentrations are usually very low, MPs may have a tendency to agglomerate. This increases the chance that particles may partly overlap. Another more common problem is that biological remnants cover parts of MPs. As the current machine learning model does not support the identification of mixed spectra, overlapping regions cannot be correctly classified by the RDF model. By using the particle editor, however, it is fairly easy to use the underlying visual image to manually define particle contours correctly. A possible bias may thus be corrected by the researcher.
- Due to their stiffness, fibers may not lie flat on top of the filter surface, and therefore, parts that stick out may not be within the focal plane of the detector. As a result, a single fiber may be detected as a series of disconnected fragments (see Figure 1a, for an example). Again this issue may be corrected by using the visual image to connect the fragments with additional class pixels in between. According to Primpke et al.,^41^ covering the sample with a BaF_2_ window ensures that fibers are arranged within the focal plane of the microscope. This might be an alternative approach if large quantities of microfibers have to be analyzed.
- Total absorption (TA) can be another prominent problem in transmission measurements if MPs exceed a certain thickness. Figure 1c and d shows particles where TA hampers their correct identification. For the less severely affected spectra, the TA effect may still allow polymers to be identified if sufficient information on peak positions is left. The employed RDF model has been specifically trained to allow for a classification of such spectra. Nevertheless, there are particles which can only be partly detected which again requires a manual user intervention using the visual image in conjunction with the particle editor.

### Cross Validation and Performance Measures

The confusion matrix which is depicted in Figure S1 and Table S2 shows that there are only a few cases where a certain polymer type has been assigned to a wrong class. On the other hand there are more cases of wrong predictions regarding polymers and matrix residuals (see entries for class “Other”). Not surprisingly, this classification problem is much more difficult to solve for the RDF algorithm, as matrices are very heterogeneous, in general.

Table 1 and Table S1 further summarize the confusion matrix in the form of performance measures.^26^ Please consider that the given measures only reflect the performance of the algorithm within the boundaries where experts were still able to determine a ground truth. We would also like to state that a comparison with other algorithms based on the herein published performance measures would be an invalid comparison, as the test data sets need to be the same. See Demšar^42^ on how to compare classifiers over multiple data sets.
