# Supplementary material for: Computer-Assisted Analysis of Microplastics in Environmental Samples Based on μFTIR Imaging in Combination with Machine Learning
Source: Environ Sci Technol Lett. 2021 Dec 9;9(1):90–5. doi: 10.1021/acs.estlett.1c00851 (PMC8757466; doi:10.1021/acs.estlett.1c00851)
Supplement: Supplementary file 1 — ez1c00851_si_001.pdf [file ez1c00851_si_001.pdf]

Supporting Information
Computer-assisted analysis of microplastics in environmental samples based on μFTIR imaging in combination with machine learning

by

Benedikt Hufnagl,[a,b,*] Michael Stibi,[b] Heghnar Martirosyan,[c] Ursula Wilczek,[c] Julia N. Möller,[c] Martin G. J. Löder,[c] Christian Laforsch[c] and Hans Lohninger[a]

[a] *Institute of Chemical Technologies and Analytics; Vienna University of Technology; A 1060 Vienna, Austria benedikt.hufnagl@tuwien.ac.at; https://orcid.org/0000-0002-1470-5787*

[b] *Purency GmbH, Walfischgasse 8/34, A 1010 Vienna, Austria*

[c] *Department of Animal Ecology I and BayCEER; University of Bayreuth; D 95440 Bayreuth, Germany; D 95440 Bayreuth, Germany*

Enzymatic preparation scheme

The preparation scheme described by Löder et al. [1] starts with splitting the sample in two fractions using a 500 μm sieve. The larger fraction is sorted manually using a stereo microscope followed by a spectroscopic identification by means of attenuated total reflectance FTIR (ATR-FTIR). The smaller fraction below 500 μm is then digested using different enzymes and hydrogen peroxide. A final density separation by means of a zinc chloride solution is applied to remove residual mineral particles. The sample is then filtered through an aluminum oxide filter (Anodisc 0.2 μm pore size, 10 mm diameter) which is the sample carrier for the spectroscopic analysis.

According to Löder et al. [1] the protocol may be applied to a wide range of different environmental matrices and requires about 16 days while the actual handling time per sample is only about 4 hours. The protocol therefore allows many samples to be processed in parallel and can be optimized for certain matrices as applying all steps will not be necessary in general.

Table S1: Multi-class performance measures. Definitions and scripts for computing the given performance measures can be found in Ballabio et al. [2].

Performance measure	Value
accuracy	0.9766
Kappa	0.9690
ner	0.9710
Pr	0.9761
GSn	0.9708
GPr	0.9760
GmM	0.9846
V	0.9736
VM	0.9735
F	0.9736
FM	0.9734
JM	0.9695
sInd	0.9792
EMCC	0.9690
AUNU	0.9847
AUNP	0.9831
AU1U	0.9986

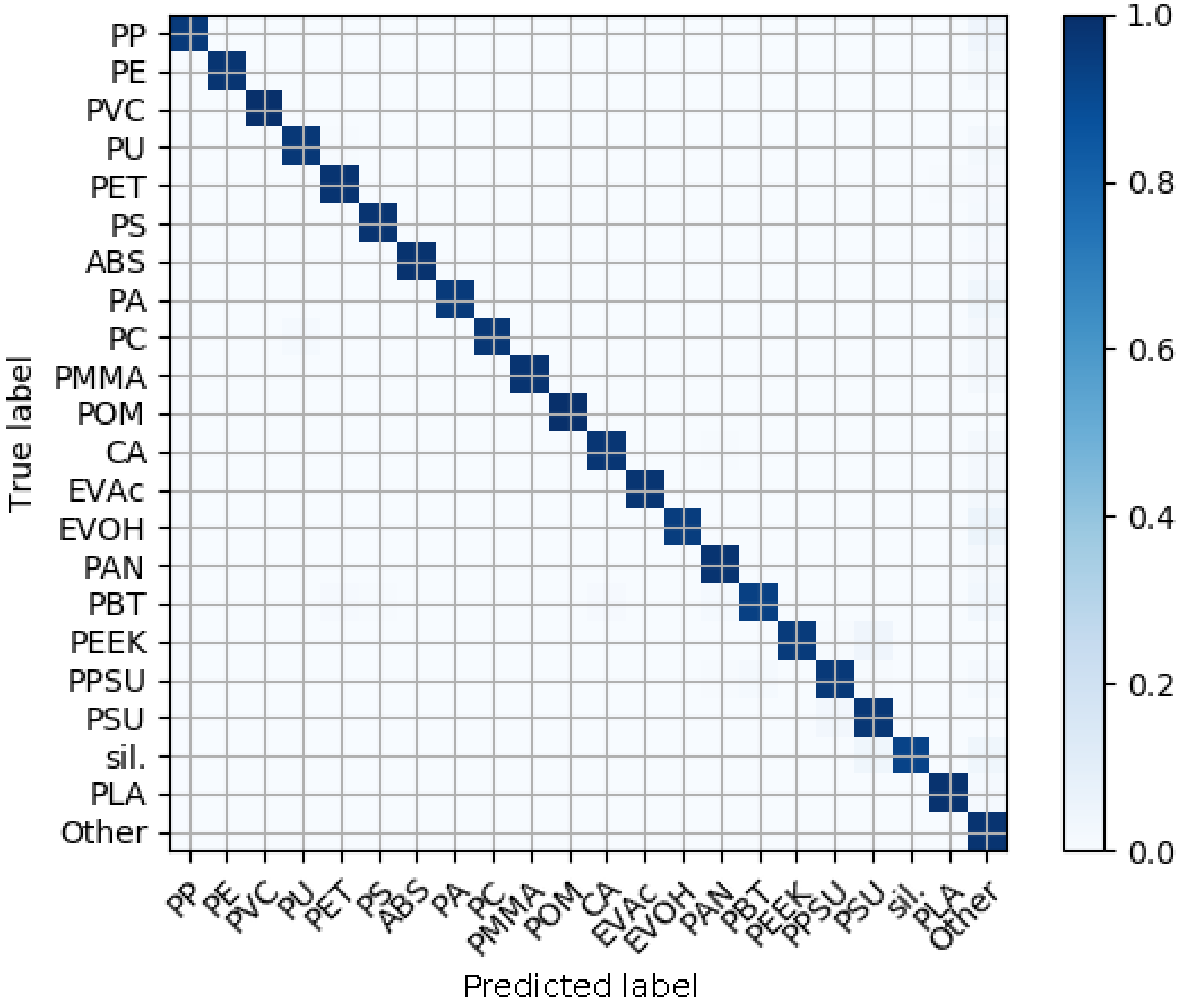

Figure S1: Normalized confusion matrix of the Monte Carlo cross validation.

Table S2: Original confusion matrix without normalization applied.

	PP	PE	PVC	PU	PET	PS	ABS	PA	PC	PMMA	CA	EVAc	EVOH	PAN	PBT	PEEK	POM	PPSU	PSU	silicone	PLA	Other
PP	1206	0	0	0	0	0	0	0	0	0	0	0	0	0	0	2	0	0	0	0	0	52
PE	1	1272	0	0	0	0	0	0	0	0	0	0	0	0	0	0	0	0	0	0	0	27
PVC	0	0	480	0	0	0	0	0	0	0	0	0	0	0	0	0	0	0	0	0	0	0
PU	0	0	0	619	3	0	0	1	0	2	1	0	0	0	0	0	0	0	0	0	0	14
PET	0	0	1	0	1002	0	0	0	0	0	0	0	0	0	0	2	0	0	0	0	4	11
PS	0	0	0	0	0	707	0	1	0	0	0	0	0	0	0	2	0	0	0	0	0	10
ABS	0	0	1	0	0	1	355	0	0	0	0	0	0	0	0	0	0	0	0	0	0	3
PA	0	1	0	0	0	0	0	1015	3	0	0	0	0	0	0	0	0	0	0	0	0	41
PC	0	0	0	4	0	0	0	0	330	0	0	0	0	0	0	0	0	0	0	0	0	6
PMMA	0	0	0	0	0	0	0	0	0	963	0	0	0	0	0	0	0	0	0	0	0	17
CA	0	0	0	0	0	0	0	0	0	0	300	0	0	0	0	0	0	0	0	0	0	0
EVAc	0	0	0	0	0	1	0	0	0	0	0	370	0	0	2	0	0	0	0	0	0	7
EVOH	0	0	0	0	0	0	0	0	0	0	0	0	665	0	0	0	0	0	0	0	0	15
PAN	0	0	0	0	0	0	0	0	0	0	0	0	0	284	0	0	0	0	0	0	0	16
PBT	0	0	0	0	0	0	0	0	0	0	0	0	0	0	393	0	0	0	0	0	0	7
PEEK	0	0	0	0	3	2	0	0	0	0	0	3	0	0	5	337	0	0	0	0	0	10
POM	0	0	0	0	0	0	0	0	0	0	0	0	0	0	0	0	286	2	12	0	0	0
PPSU	0	0	0	0	0	0	0	0	0	0	0	0	0	0	2	4	0	328	2	0	0	4
PSU	0	0	0	0	0	0	0	0	0	0	0	0	0	0	0	0	0	8	291	0	0	1
silicone	0	0	0	0	0	0	0	0	0	0	0	0	0	0	0	0	1	0	10	259	0	10
PLA	0	0	0	0	0	0	0	0	0	0	0	0	0	0	0	0	0	0	0	0	730	10
Other	35	33	8	15	19	11	2	19	7	15	1	1	20	1	3	2	0	5	4	3	10	11306

References

1. Löder, M. G. J.; Imhof, H. K.; Ladehoff, M.; Löschel, L. A.; Lorenz, C.; Mintenig, S.; Piehl, S.; Primpke, S.; Schrank, I.; Laforsch, C.; Gerdts, G. Enzymatic purification of microplastics in environmental samples. *Environmental science & technology* **2017**, *51*, 14283–14292.

2. Ballabio, D.; Grisoni, F.; Todeschini, R. Multivariate comparison of classification performance measures. *Chemometrics and Intelligent Laboratory Systems* **2018**, *174*, 33–44.

3. Hufnagl, B.; Steiner, D.; Renner, E.; Löder, M. G. J.; Laforsch, C.; Lohninger, H. A methodology for the fast identification and monitoring of microplastics in environmental samples using random decision forest classifiers. *Analytical Methods* **2019**, *11*, 2277–2285.

4. Primpke, S.; Wirth, M.; Lorenz, C.; Gerdts, G. Reference database design for the automated analysis of microplastic samples based on Fourier transform infrared (FTIR) spectroscopy. *Analytical and bioanalytical chemistry* **2018**, *410*, 5131–5141.

PP	PE	PVC	PU	PET	PS	ABS
PA	PC	PMMA	POM	CA	EVAc	EVOH
PAN	PBT	PEEK	PPSU	PSU	silicone	PLA

Figure S2: Associated class colors for the respective 21 polymer types.

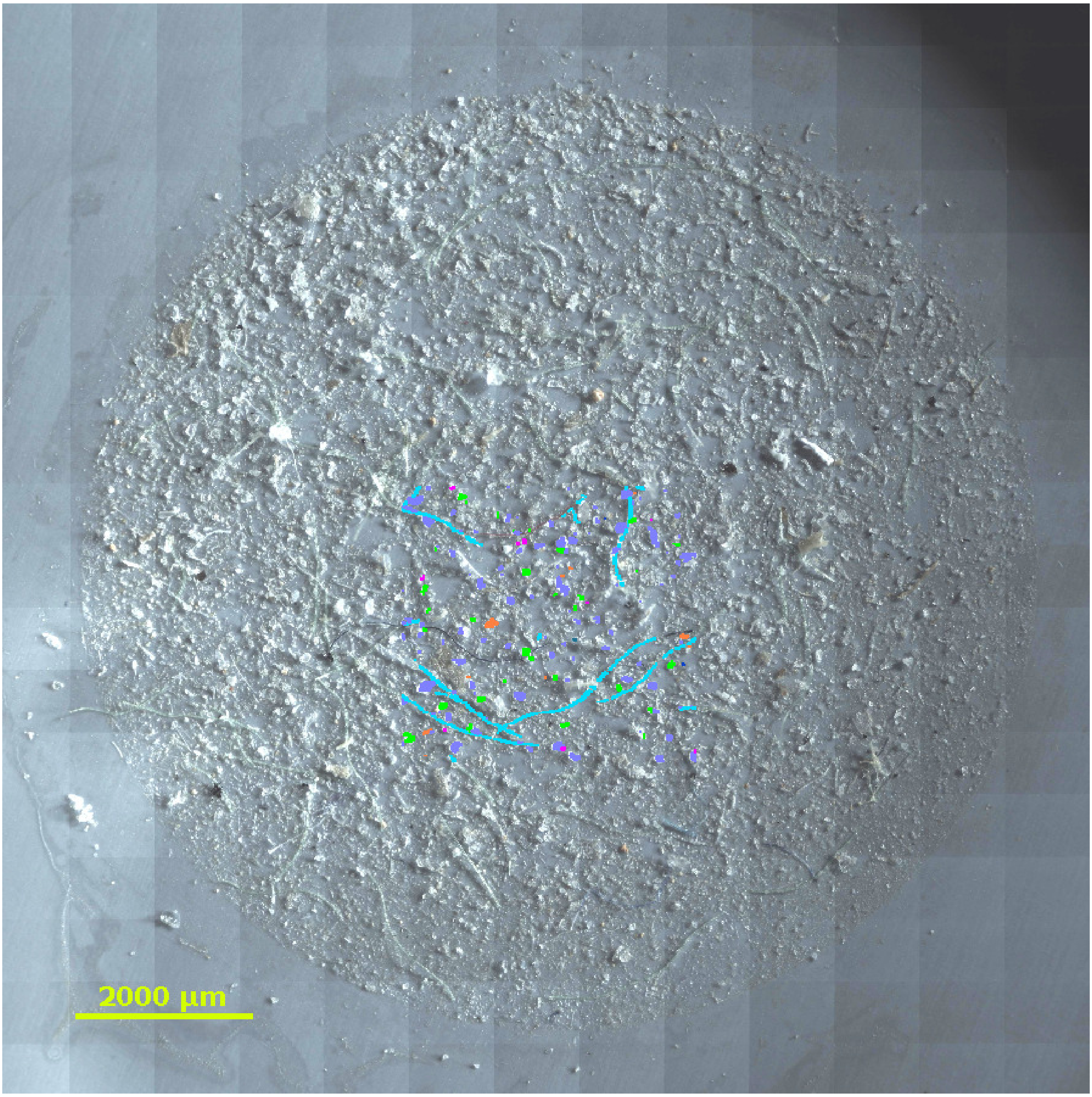

Figure S3: Plankton sample adapted with permission under a Creative Commons Attribution 3.0 Unported License from Hufnagl et al.[3], Copyright 2019 The Royal Society of Chemistry, original microscope image superimposed with new classification result, DOI: 10.1039/C9AY00252A The original hypercube covered the entire sample area but was trimmed. See figure S2 for the corresponding class colors.

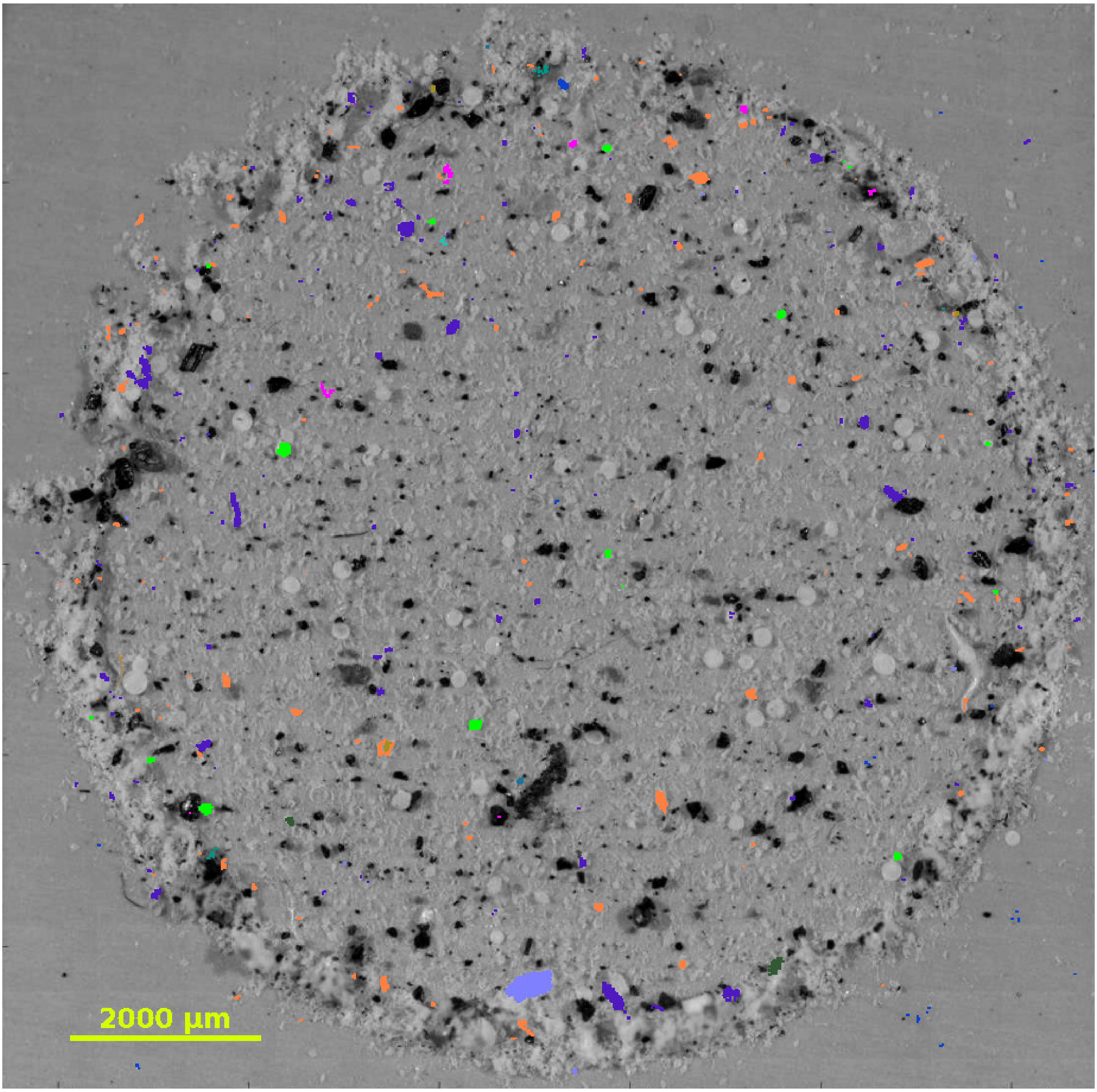

Figure S4: Reference sample 'RefEnv1' adapted with permission under a Creative Commons Attribution 4.0 International License from Primpke et al. [4], Copyright 2018 Springer Nature, original microscope image superimposed with new classification result, DOI: s00216-018-1156-x. See figure S2 for the corresponding class colors.

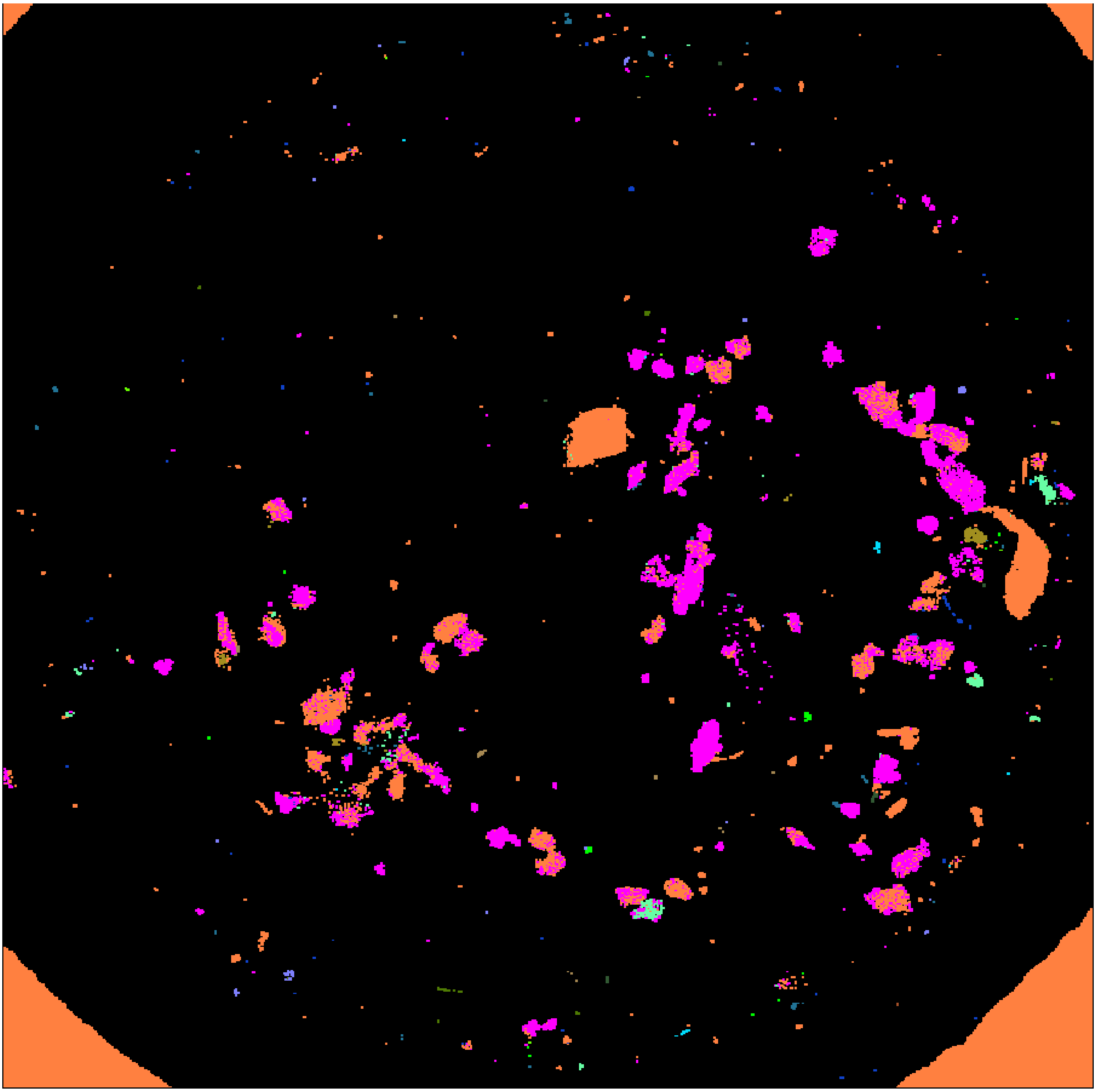

Figure S5: Reference sample 'RefEnv2' adapted with permission under a Creative Commons Attribution 4.0 International License from Primpke et al. [4], Copyright 2018 Springer Nature, derivative work showing the classification result when the RDF is applied to the dataset, DOI: s00216-018-1156-x. See figure S2 for the corresponding class colors.

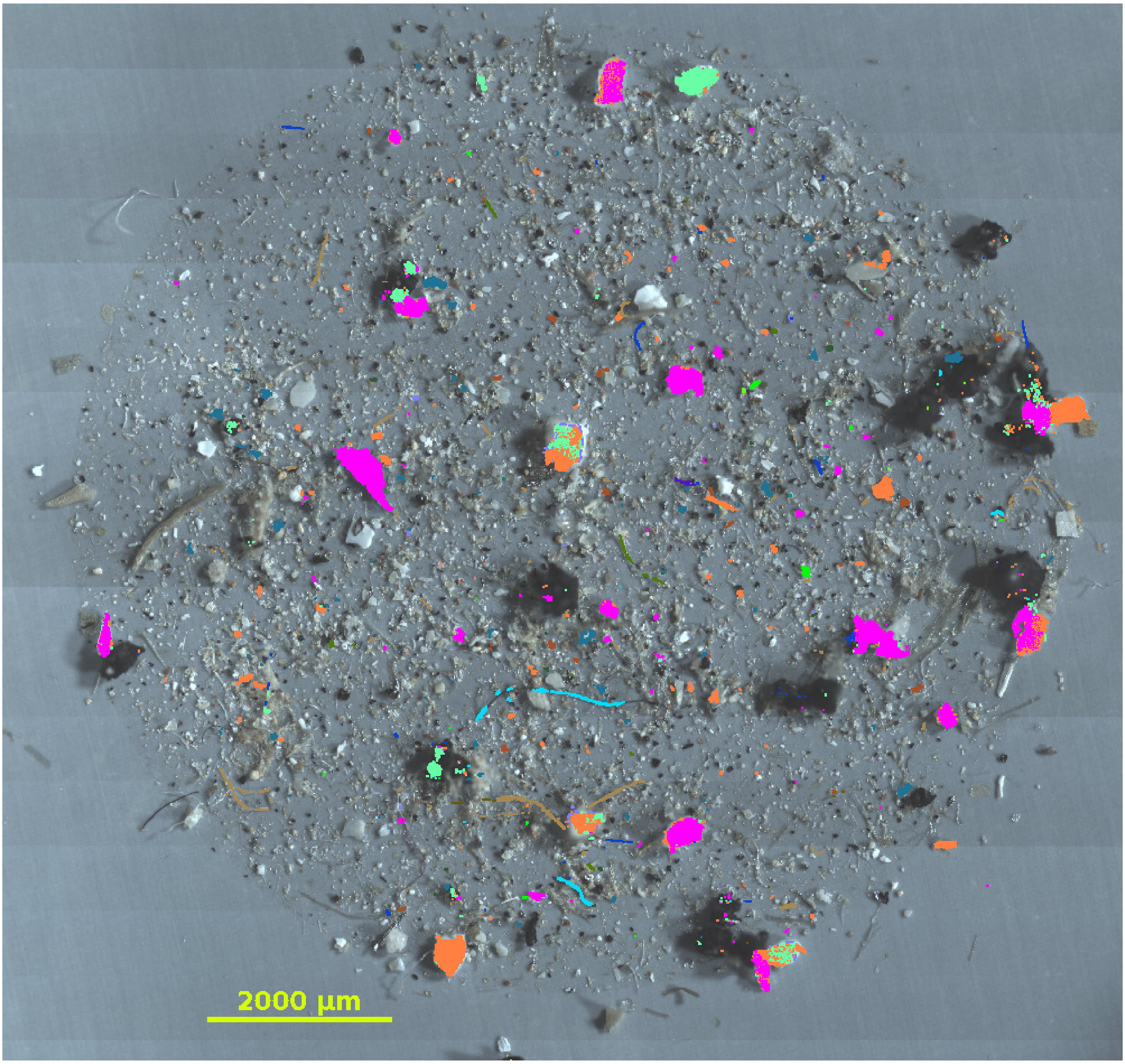

Figure S6: Waste water treatment plant outlet sample. See figure S2 for the corresponding class colors.

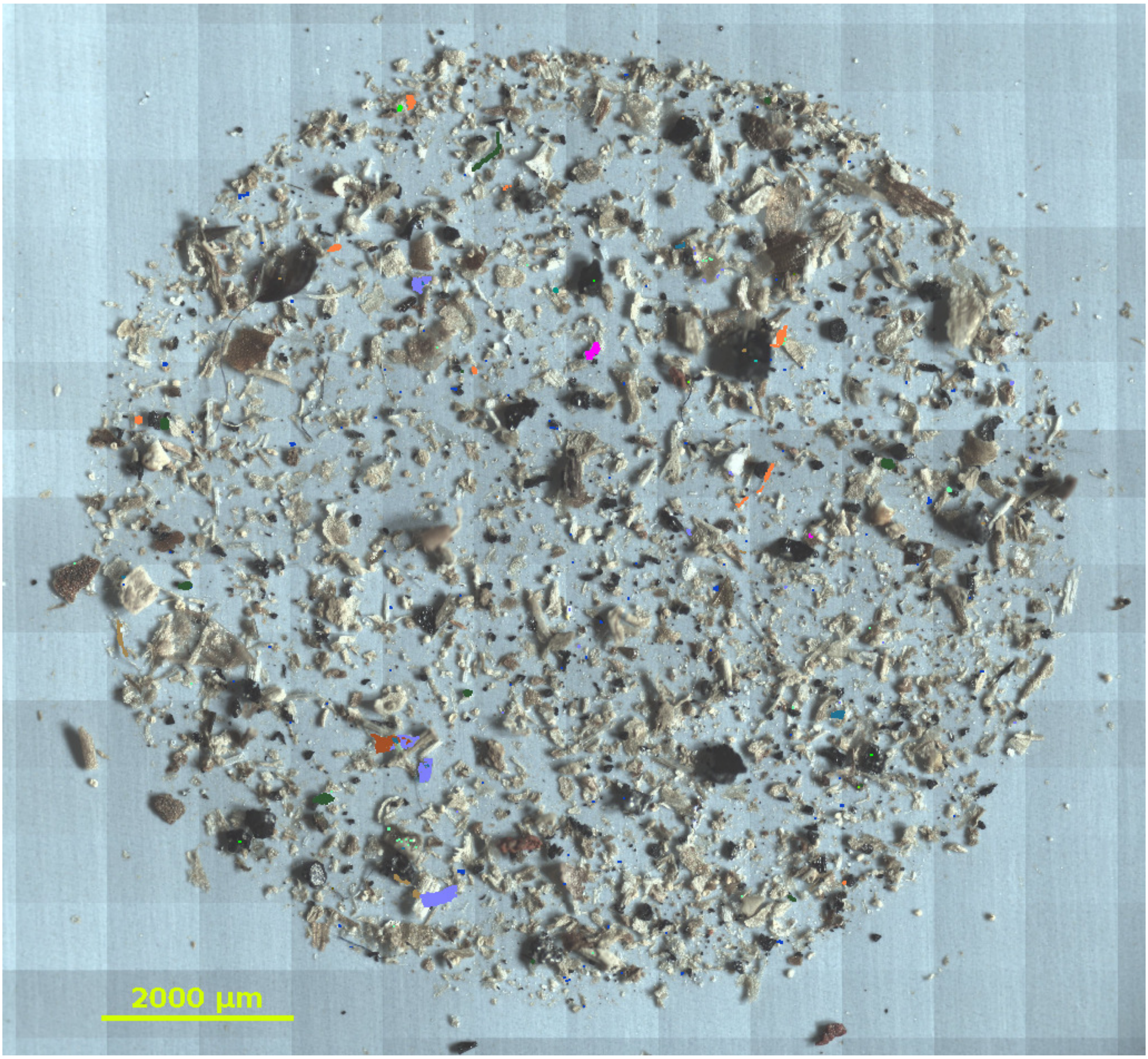

Figure S7: Deep sediment sample. See figure S2 for the corresponding class colors.

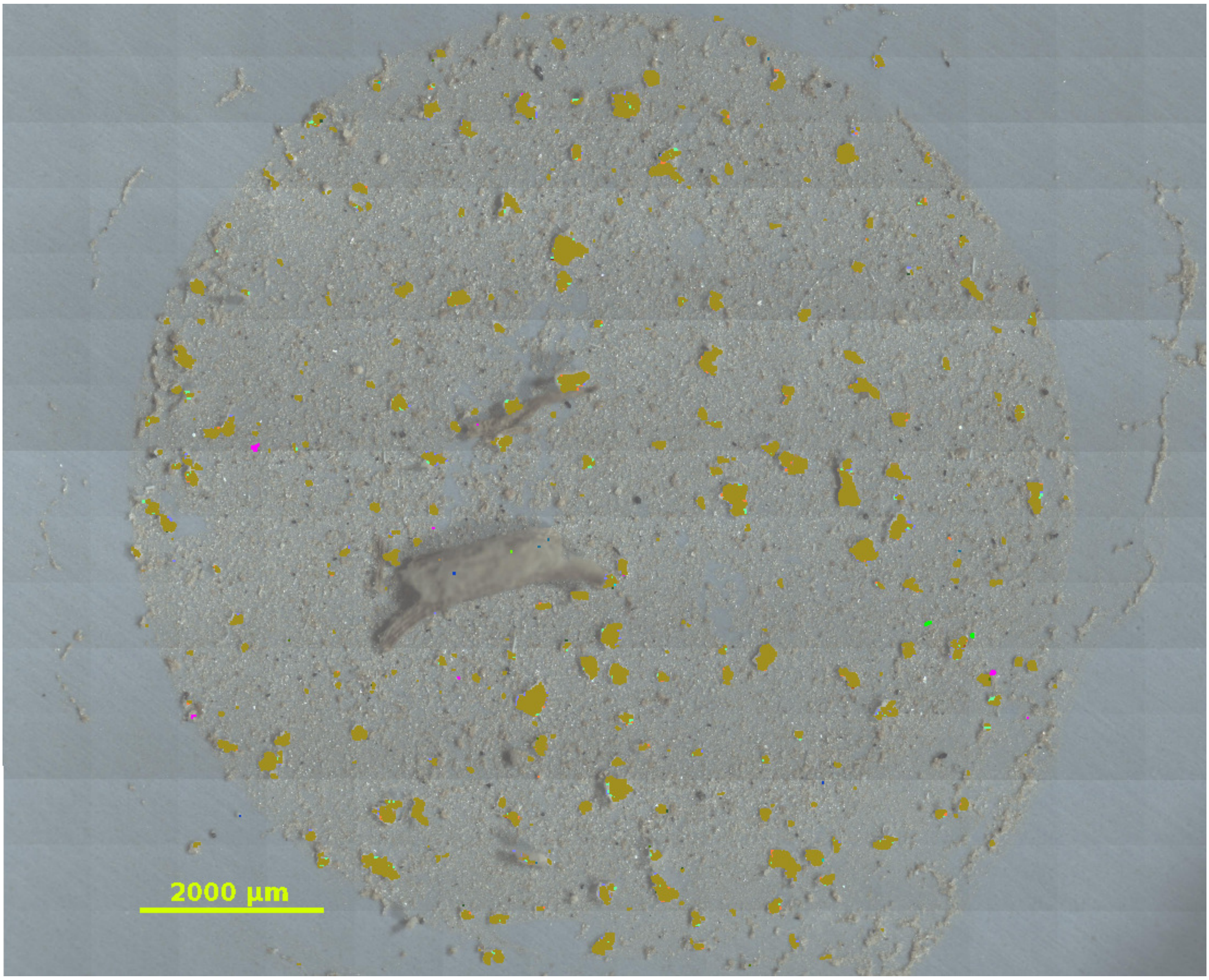

Figure S8: Soil sample. See figure S2 for the corresponding class colors.

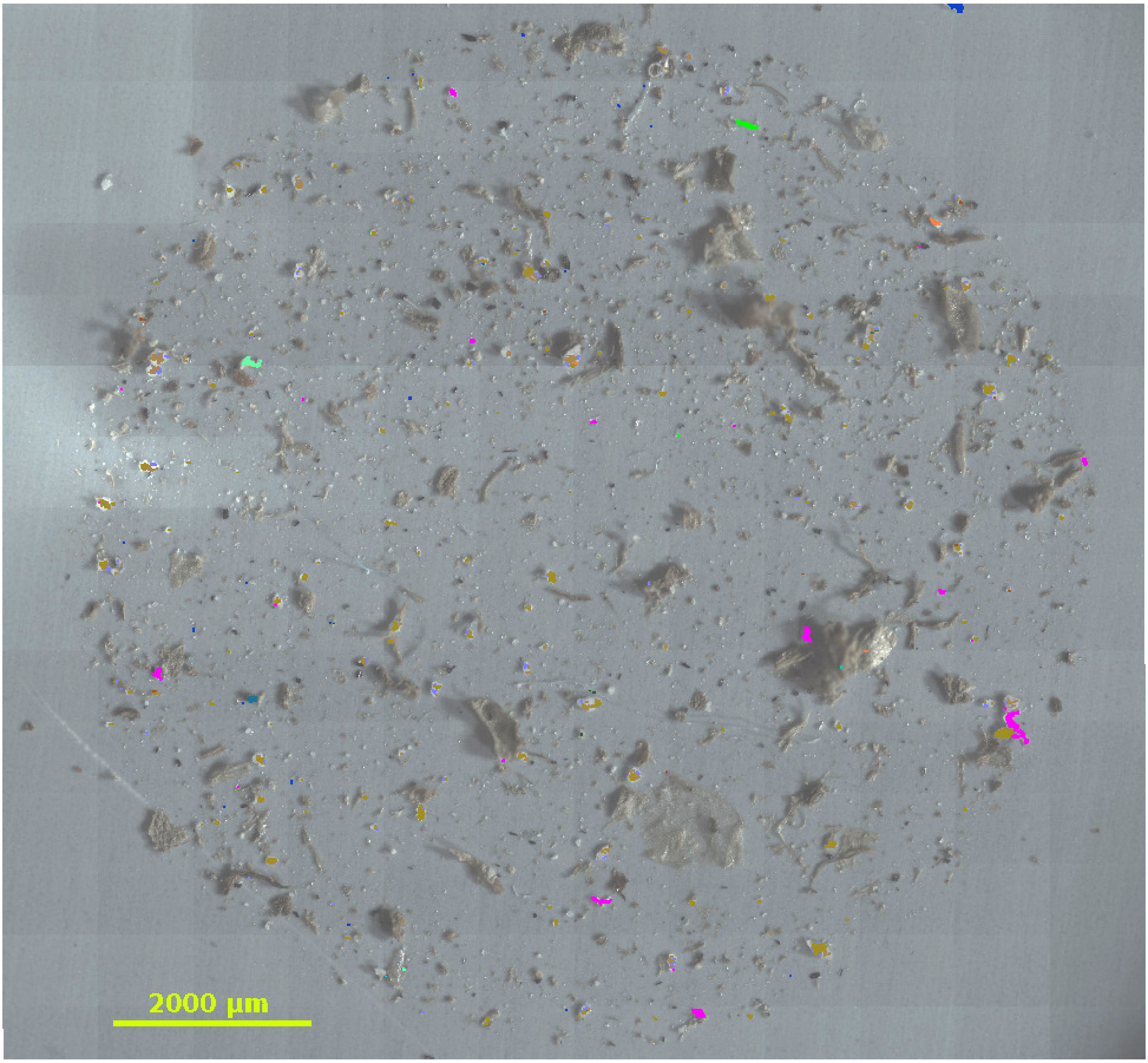

Figure S9: Compost sample. See figure S2 for the corresponding class colors.

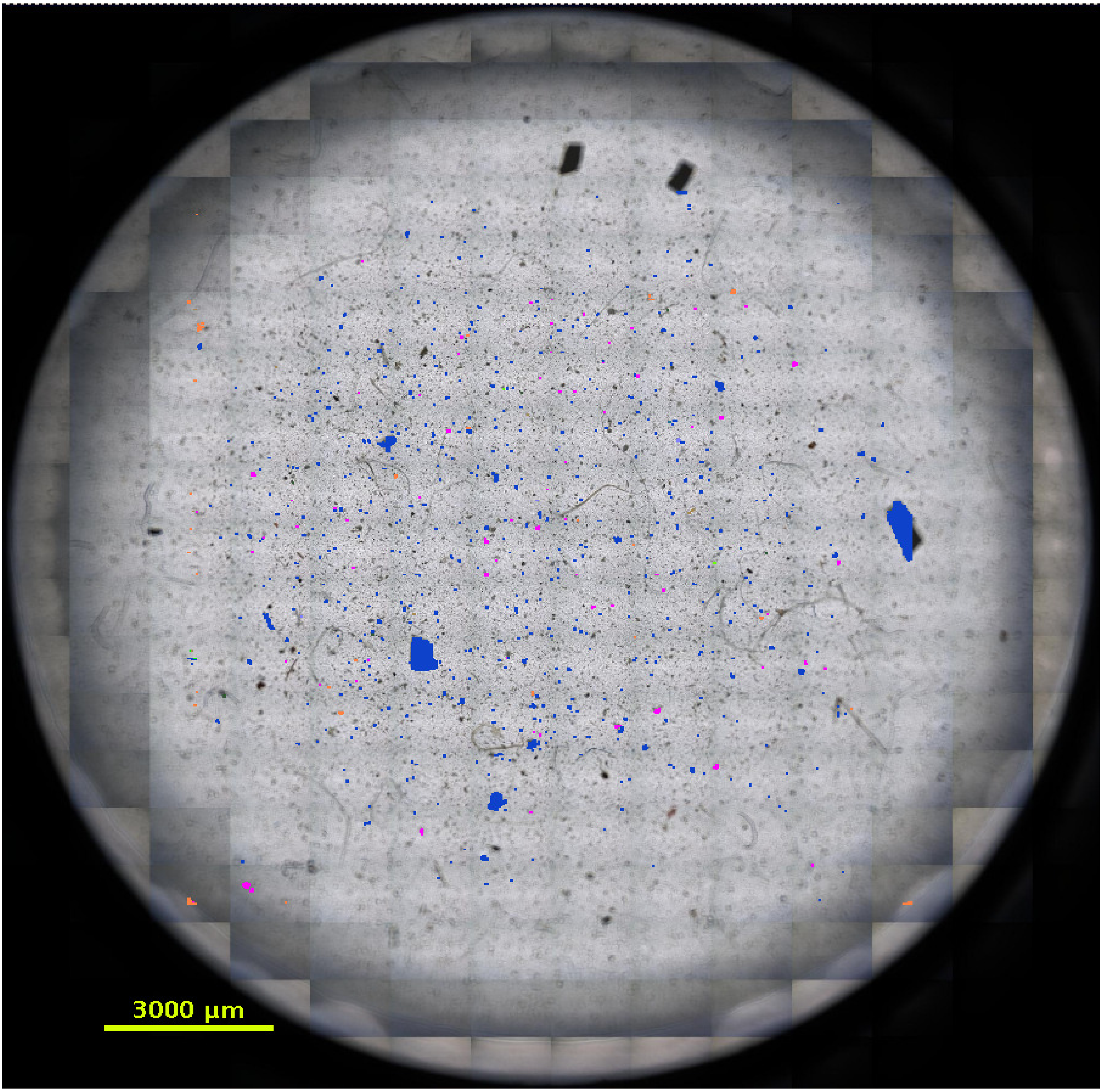

Figure S10: Sea salt sample measured with Bruker LUMOS II. See figure S2 for the corresponding class colors.

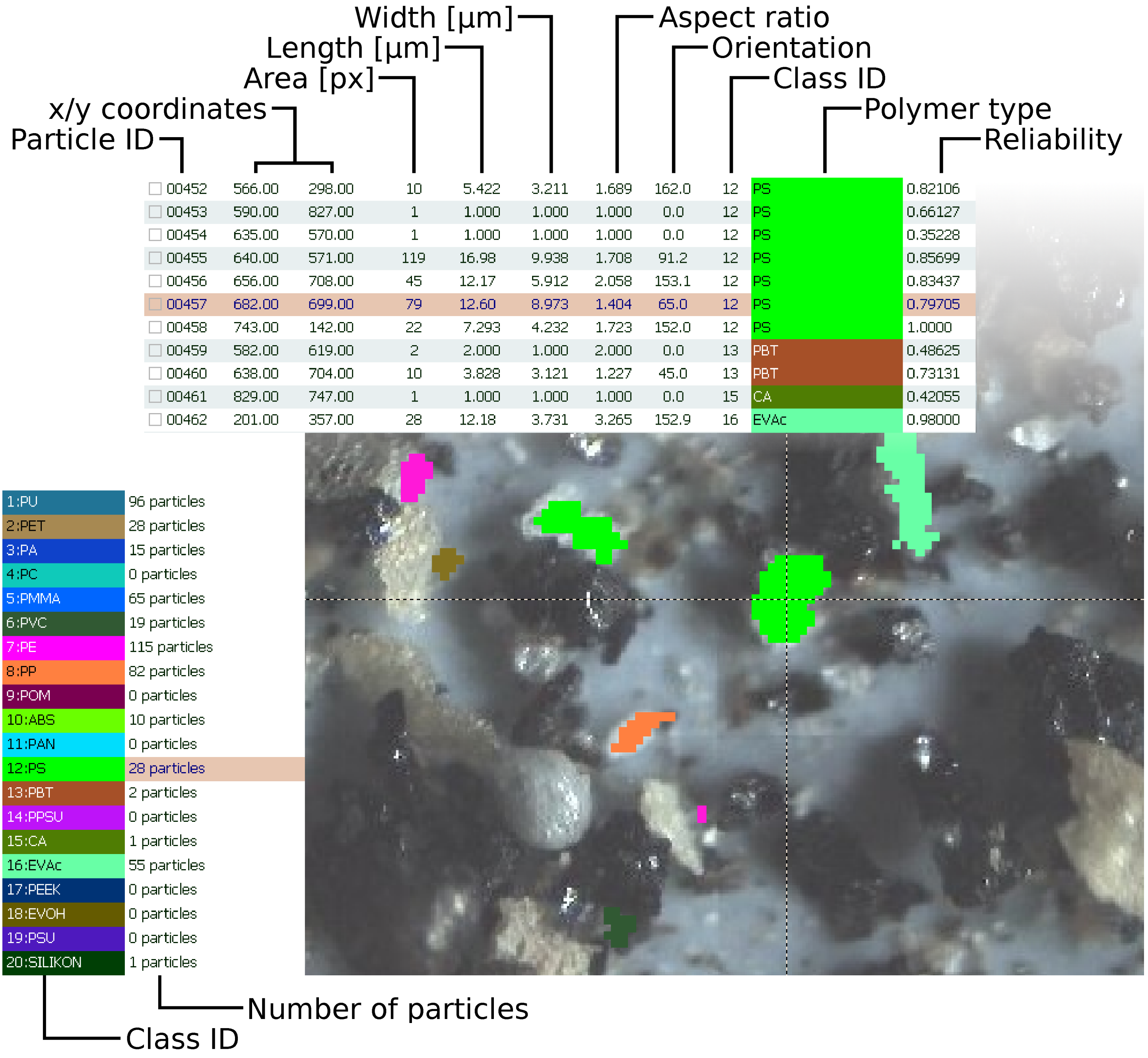

Figure S11: Selected elements of the graphical user interface.

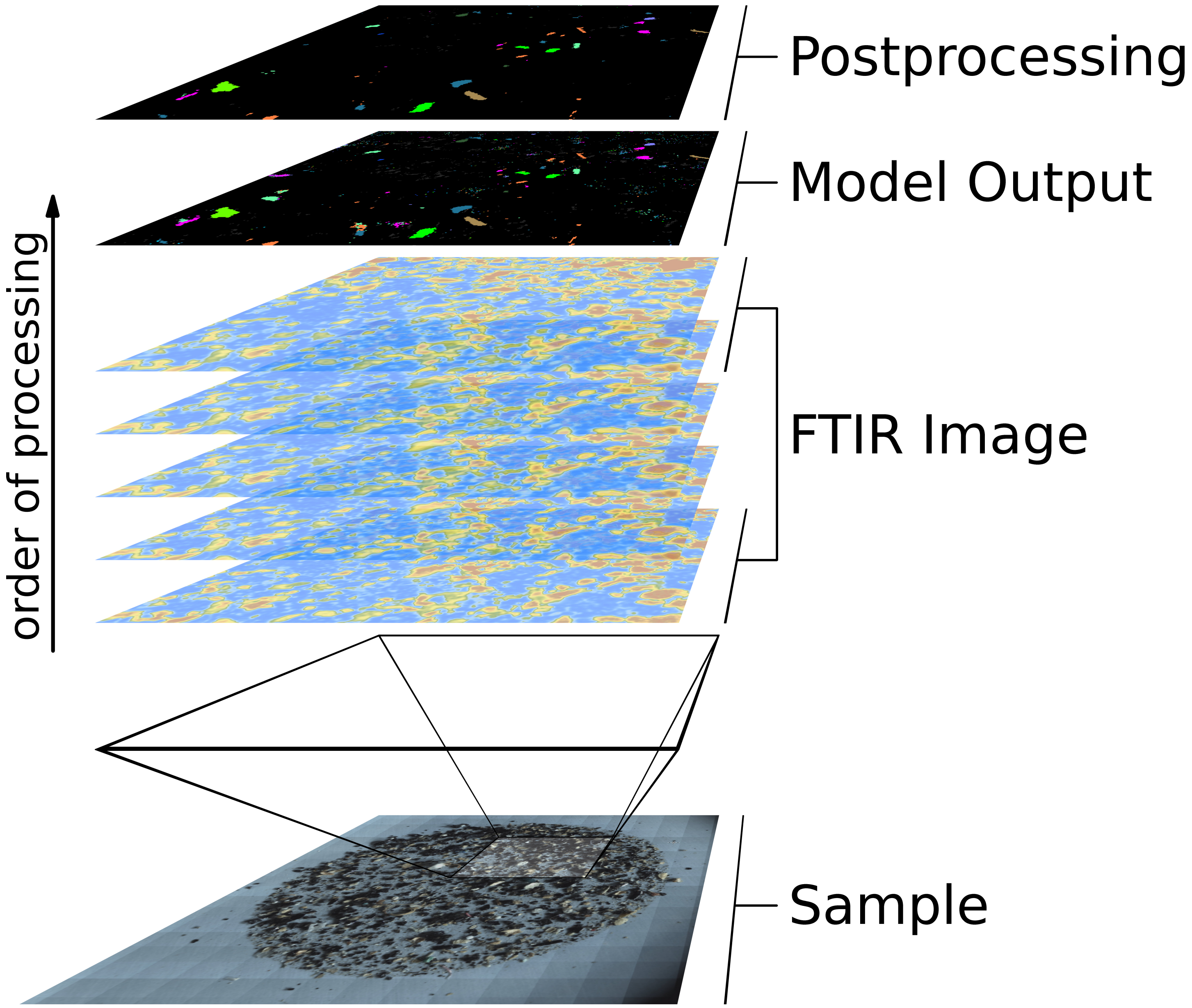

Figure S12: Data acquisition and processing of the Bayreuth Microplastics Finder approach. In this illustration the sample surface is mapped using an FPA-based FTIR imaging spectrometer which produces a high-resolution chemical image. Using a statistical model different microplastics are detected to form an initial model output. In a subsequent step this result is postprocessed and forwarded to the particle characterisation tool which then allows size distributions to be estimated.
